# Innovations of renal replacement techniques at the intersection of clinical trial and real-world data

**DOI:** 10.1007/s11255-023-03738-3

**Published:** 2023-08-15

**Authors:** Helmut Schiffl

**Affiliations:** 1 Department of Medicine IV, Hospital of the LMU Munich, Ziemssenstr. 3, 80336 Munich, Germany; 2 Medizinische Klinik und Poliklinik IV, Universitätsklinikum der LMU München, Ziemssenstr. 3, 80336 Munich, Germany

Editor,

End-stage kidney disease (ESKD) patients on standard haemodialysis (HD) continue to bear a high burden of morbidity, frequent hospitalizations, shortened life expectancy and low health related quality of life. The status quo of current dialysis care is suboptimal. There are unmet needs for innovative renal replacement techniques to reduce the residual uremic syndrome. Numerous observational studies, few randomized-controlled trials, and meta-analyses focusing on the superiority of advanced renal replacement therapies [haemodiafiltration (HDF), expanded haemodialysis] provided conflicting results regarding hard patient-related outcomes, especially in terms of mortality [1].

New data of the CONVINCE open-label, multi-center, randomized-controlled trial show a lower risk of all-cause mortality (hazard ratio 0.77) among ESRD patients receiving high-volume post-dilution online HDF than among those who were receiving high-flux HD. The reduction of the mortality risk in high-dose HDF patients resulted from less infection-related fatalities, including COVID-19. The risk of death from fatal or non-fatal cardiovascular events was similar in the HDF and HD group [2].

Without doubt, the CONVINCE trial has all the ingredients of a pragmatic landmark study and it represents a major step forward in reducing the excess mortality of ESKD patients. However, CONVINCE has weaknesses of study design and methodology that may influence the results, conclusions, and generalizability.

The trial recruited study participants who were healthier and more motivated patients than the real-world dialysis patient population. The trial deliberately excluded ESKD patients commencing dialysis during the 90-day period with excessive high mortality risk, subjects with comorbidities that would limit lifespan, or patients who were not willing to have dialysis sessions with duration of more than 4 h three times a week or were non-adherent to medication [3].

The risk of death analysis in pre-defined subgroups yielded—at least in part—unexpected results. The death rate per 100-person year was lower in patients with a dialysis vintage less than 2 years or patients older than 65 years. It was unrelated to residual urinary output. Regrettably, data on residual diuresis were available in only 11% of participants. These counter-intuitive findings suggest residual confounding by uncontrolled or unknown variables.

Moreover, there are inherent limitations of the endpoint adjudication process of clinical events (causal association of the event with RRT). Clinicians cannot distinguish between deaths due to COVID-19 or deaths of patients with COVID-19 without postmortem autopsy. There is missing information on the pathogens (primary or secondary bacterial infections), nor the course and treatment of fatal infections. Of great importance, the biological mechanisms of the greater susceptibility of the high-flux patients remain speculative (compromised immunocompetence due to less elimination of middle-sized uremic molecules or disruption of intestinal barrier during hypotensive episodes).

The authors used total (per session) and not standardized convection volumes (body surface or body weight). The centers participating in the CONVINCE trial had experience with online HDF.

The CONVINCE trial demonstrates that a durable convection volume of more than 23 l/ session is feasible in most ESKD patients, and that high-dose post-dilution HDF reduces the mortality risk of subgroups of ESKD patients requiring maintenance RRT. Clinical trial results combined with real-world cohort science data will promote the uptake of high-dose post-dilution online HDF.
